# Recreational athletes during downhill-mountain biking (DMB) show high incidence of upper extremity fractures in combination with soft-tissue injuries

**DOI:** 10.1038/s41598-024-54774-7

**Published:** 2024-02-20

**Authors:** Franziska Lioba Breulmann, Claudia Krenn, Lukas Fraißler, Harald Kindermann, Michael Gattringer, Michael Stephan Gruber, Sebastian Siebenlist, Georg Philipp Mattiassich, Martin Bischofreiter

**Affiliations:** 1 Department of Sports Orthopedics, Klinikum rechts der Isar, Technical University of Munich, 81675 Munich, Germany; 2 Department of Orthopedics and Traumatology, Klinik Diakonissen Schadming, 8970 Schladming, Austria; 3 Teaching Hospital of the Paracelsus Medical University Salzburg, 5020 Salzburg, Austria; 4 Department of Marketing and Electronic Business, University of Applied Sciences Upper Austria, 4400 Steyr, Austria; 5 Department of Orthopedic Surgery, Ordensklinikum Linz, Barmherzige Schwestern, Vinzenzgruppe, Center of Orthopedic Excellence, 4020 Linz, Austria

**Keywords:** Epidemiology, Risk factors, Orthopaedics

## Abstract

Downhill-mountain biking (DMB) is a high-risk sport and often leads to several injuries, especially in non-professional athletes. We retrospectively analyzed the most common injuries and profiled the injury mechanism. Until now, there is no such analysis of injuries by non-professional mountain bike athletes. We collected patient data from patients who suffered from an injury during DMB. The inclusion criteria were (1) injury during the summer season of 2020 and 2021, (2) injury during off-road and downhill mountain bike sports activity, and (3) treatment at the Department of Traumatology of the Klinik Diakonissen Schladming. Patient data was analyzed regarding the type of injury, location of the injury, patient age and gender of the patients. Most patients with injury are at the age of 26–35. Second most are between 36 and 71 years old. The type of injury differs between age and gender. Mostly upper-extremity injuries occur with a high probability of shoulder injuries. In the elderly patients, we found additional injuries of the thorax and chest. To conclude, most common types of injuries are soft-tissue injuries, often in combination with fractures. The risk for injuries is higher for recreational athletes with different injury characteristics than professional athletes.

## Introduction

Downhill-mountain biking (DMB) is a rapidly growing sport and an activity at high risk of injuries. The demographics and injury epidemiology, as well as mechanisms, are not fully understood.

Off-road mountain biking appeals to individuals of many different ages. This sport remains popular, particularly among young and middle-aged men. The mean age is reported to be 25^1–3^. With increasing popularity and speed, the number of injuries increases and the mechanisms need attention to better prevent injuries.

In 1995, Pfeiffer et al. described an increase in the injury rate among competitive riders, from 0.2 to 0.39% and 0.3% for recreational riders^4^. Moreover, 88% of mountain bike riders were found to suffer from some kind of injury throughout their career. Off-road mountain biking athletes showed an overall injury risk rate of 0.6% per year and one injury per 1000 h of biking^1^. A slippery road surface, excessive speed, and personal fitness are risk factors for injuries^1^. Pfeiffer et al. showed that off-road cyclists sustain more fractures, dislocations, and concussions than road cyclists^4^.

The injuries sustained during DMB are described as being more often from falling forward over the handlebars and less often from falling to the side (65% vs. 25%)^5^. The kinds of injuries are different depending on the injury mechanism. Falling forward over the handlebars leads to more severe injuries, while falls to the side lead to more injuries of the lower extremities^5^. Overall, most injuries sustained during off-road mountain biking are characterized as minor, while Chow et al. found that 26% of injured individuals required professional medical care and 4.4% had to be treated in hospital^2^. Previously, Gaulrapp et al. described 75% of the injuries as minor injuries such as skin wounds or contusions, while 10% required hospital treatment^1^.

There are few studies focusing on the mechanism and injury characteristics of off-road cyclists. Kronisch et al. studied off-road mountain bike injuries in a prospective manner to describe the mechanisms of injury. They described a possible correlation between injury mechanism and injury severity^3,6^.

Most data are only reported in professional athletes or from competing off-road events. In addition, some case reports or questionnaires have been published that show different kinds of injuries among recreational athletes than in professional athletes. Indeed, 50–90% of all recreational athletes reported any kind of injury during DMB^1^. We hypothesize that the level of sports activity might influence the injury profile and number of injuries.

This study aims to better understand the injury mechanisms, injury types, and location and severity of injuries suffered by off-road and downhill mountain bike riders. Until now, there has been no demographical analysis of injury types from DMB. We hypothesize that there is a clear profile of injuries in recreational athletes. Injuries of the upper extremities might be more common than of the lower extremities, and more severe injuries of the thorax and head are rare.

## Materials and methods

### Data collection and analysis

We performed a retrospective analysis of patients who suffered from a downhill mountain bike injury. For retrospective analysis, we collected data from patients who suffered from an injury during mountain biking activity and underwent treatment at the trauma department. All investigations were performed in accordance with the Declaration of Helsinki and after the approval of the local ethics committee of the Medical University of Graz (Project No. 33-498).

The inclusion criteria were (1) injury during the summer season (May–November) 2020 and 2021; (2) injury during DMB; and (3) treatment in the trauma department of our hospital, a mountain-resort-based medical center in Austria. We excluded patients who suffered from an injury but underwent treatment in a different hospital. Reasons for no treatment in our hospital after an injury are multiple: some patients prefer treatment in their hometown hospital if the injury does not need immediate treatment, other severe injuries, e.g. severe head trauma with intracerebral bleeding, spine or thoraces trauma, are sometimes immediately transported to another trauma center close. Numbers of patients not being treated in our hospitals are not available. The patient data were then analyzed regarding the type of injury, the location of the injury, the patient’s age, and the gender of the patient. Additionally, we collected data from the Ski Lift Resort to compare the number injuries to the total amount of tickets sold for the lift and the number of rides with the lift.

### Demographic data extraction

We collected demographic data about each patient from the patient data system of the hospital. The collected data were anonymized. The type of data extracted included the age of the patient, sex, type of arrival at the hospital (walking, by ambulance), type of injury and localization (which extremity, left/right), and whether surgery was performed or recommended. The data for the number of lift cards sold and the number of lift rides were provided by the Lift Resort in Schladming, Austria.

### Statistical data analysis

The analysis was performed according to the Checklist for Statistical Assessment of Medical Papers (CHAMP) statement^7^. We performed descriptive analysis of the demographic data that were extracted retrospectively. For quantitative analysis, we compared the absolute number of injuries with the absolute frequency of injuries and calculated the percentage of the total amount of reported injuries. Moreover, we calculated the injuries per rides overall for each season separately. We quantitatively compared the total number of injuries in different locations, and analyzed the kinds of injuries and whether the patient suffered a fracture, ligament rupture, or joint or soft-tissue injury in the upper or lower extremities. For soft-tissue injuries, we differentiated between the size of the soft-tissue damage. We reported the total number of different fractures and joint injuries. Lastly, we performed a Pearson Chi-Square comparison with different sex and gender for different injury regions.

### Ethics approval and consent to participate

All procedures performed in studies involving human participants were in accordance with the ethical standards of the institutional and/or national research committee and with the 1964 Helsinki Declaration and its later amendments or comparable ethical standards. The study was approved by the Ethical Committee of the Medical University Graz, Austria (33-498 ex 20/21, 07.07.2021). Informed consent was obtained from all subjects involved in the study.

## Results

### Demographics: sex and age

**Figure 1:**
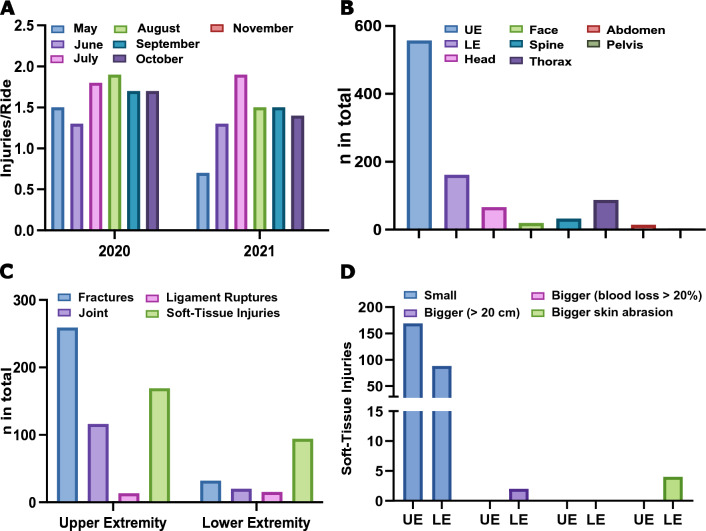
Injury overview during summer seasons in 2020 and 2021. (**A**) Injuries per year and per downhill ride over the seven months of summer season. (**B**) Locations of injuries across all patients. Injuries of the upper extremities (UE) and lower extremities (LE). (**C**) Characteristics of injuries divided for UE and LE. (**D**) Soft-tissue injuries occurring in patients suffering from UE or LE injury.

We evaluated the downhill mountain bike seasons of 2020 and 2021 from May to November in Schladming, Austria (Fig. 1). During the 2020 season, in total, 40.235 guests had a lift card for the downhill mountain bike trails and there were 180.831 single lift rides up to the start of the downhill mountain bike trails (Table 1). In the 2021 season, the number increased, with 49.423 guests in total and 226.243 lift rides. Of those guests, 936 riders suffered an injury: 437 riders in 2020 and 499 riders in 2021.

**Table 1:** Patient characteristics of age and sex.

Character	Quality	Total amount
Age	Minimum	6
	Maximum	71
	Mean	28
Sex	Male	582
	Female	68
Guests	2020	40.235
	2021	49.423
Rides	2020	180.831
	2021	226.243
Guests/Month (2020)	May	1058
	June	6533
	July	10,578
	August	12,522
	September	7031
	October	2
	November	77
Guests/Month (2021)	May	1526
	June	8309
	July	11,587
	August	14,492
	September	8617
	October	4801
	November	91
Injuries/Month (2020)	May	7
	June	38
	July	78
	August	106
	September	57
	October	21
	November	0
Injuries/Month (2021)	May	6
	June	51
	July	99
	August	96
	September	62
	October	33
	November	0
Injuries/1000 guests per month (2020)	May	6.6
	June	5.8
	July	7.4
	August	8.5
	September	8.1
	October	1.5
	November	0
Injuries/1000 guests per month (2021)	May	3.9
	June	6.1
	July	8.5
	August	6.6
	September	7.2
	October	6.9
	November	0

Total number of injuries per 1000 guests for each season (2020 and 2021).

The peak of injuries occurred in July, August, and September, which were also the months with the most visitors of the season (Table 1). In July 2020, there were 7.4 injuries per 1000 guests reported, while there were 8.5 per 1000 in 2021. For August, there were 8.5 in 2020 and 6.6 in 2021 and for September there were 8.1 injuries in 2020 and 7.2 injuries in 2021. In terms of sex, 89.9% were male and only 10.1% were women. The mean age reported was 28 years (6–71 years old). To show differences in risk for sex or age for different injury regions, we performed a chi-square test. We did not see any significant differences for sex and age (Supplement 1). Most of the patients sustained an injury to the upper extremities (UE), followed by injuries to the lower extremities (LE) (Fig. 1B). The most common injuries in the UE group were fractures, followed by soft-tissue injuries. In the LE group, soft-tissue injuries were the most commonly reported injury (Fig. 1C). Most of the soft-tissue injuries were small wounds. Larger wounds of more than 20 cm in length only occurred in two patients with lower extremity injuries (Fig. 1D).

### Upper extremity injuries

**Figure 2:**
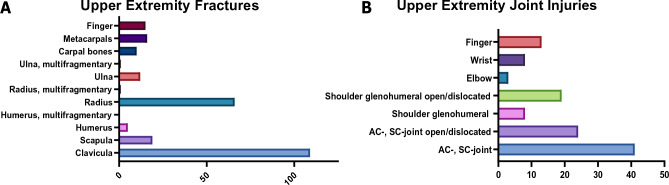
Injury characteristics in patients reporting an upper extremity injury. (**A**) Specification of the upper extremity fractures. (**B**) Specification of the upper extremity joint injuries.

Overall, 259 fractures of the upper extremities were reported, with most fractures being of the clavicula (n = 109) and the radius (n = 66). Of the radius fractures, 52 were classified as distal radius fractures, while 5 were forearm fractures and 9 were radial head fractures. Only one forearm fracture was described as an open fracture (Fig. 2).

Additionally, 116 patients suffered from a joint injury, with 41 patients showing an acromio-clavicular ligament (AC) and coraco-clavicular ligament (CC) contusion or subluxation, and 24 patients exhibiting a dislocation of the AC or CC joint. From the group of patients with an AC/CC injury, 21 were classified as Rockwood I, 17 as Rockwood II, and 17 injuries as Rockwood IV. Nineteen patients had a shoulder dislocation. In total, there were 169 small soft-tissue injuries.

### Lower extremity injuries

**Figure 3:**
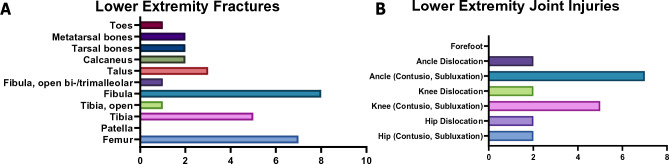
Injury characteristics among patients reporting a lower extremity injury. (**A**) Specification of the lower extremity fractures. (**B**) Specification of the lower extremity joint injuries.

From those suffering a lower extremity injury, 32 patients showed fibula (n = 8) and femur (n = 7) fractures, which were the most common fractures (Fig. 3). Twenty patients had joint injuries, with hip dislocations (n = 2) and knee dislocations (n = 2) being the most severe. Additionally, 88 riders suffered from small soft-tissue injuries.

### Rare injuries

Injuries with the involvement of the head are quite common. In total, 35 patients showed a commotio and 30 patients a contusion. Only one patient reported intracranial bleeding. For injuries involving the thorax, 45 were reported as being small soft tissue injuries. The second most common injuries of the thorax were rip fractures (n = 30), with 11 patients showing ruptures of more than three rips. Five patients suffered from a pneumothorax.

Injuries of the abdomen, pelvis, and spine were quite rare compared to the others. Twelve patients showed soft-tissue wounds of the abdomen, one patient showed a liver rupture and one patient had a testicular injury. Three patients reported a pelvis fracture following DMB. For the spine, contusions of the cervical (n = 10) and lumbar spine (n = 10) comprised the largest reported group. There were no fractures of the cervical spine reported, but some fractures of the thoracic spine (n = 5) and lumbar spine (n = 2). There were no spinal cord injuries reported.

As well, face injuries are rare. Mostly, soft-tissue injuries are reported (n = 12) and only a few fractures of the nose (n = 2).

### Injury severity

Most injuries occurred during DMB show a low severity score (Table 2). Analyzation of the National Advisory Committee for Aeronautics (NACA)-Severity Score show in total 131 injuries with a score of I, 300 with a score of II and 213 of a score of III. Only a few injuries show a higher severity with a score of IV and V. None of the injuries are more severe with the highest NACA-Scores (VI and VII).

**Table 2:** NACA-severity score.

Score	Amount of injuries at this score (n)
I	131
II	300
III	213
IV	9
V	1
VI	0
VII	0

## Discussion

### Main findings

The Mein Findings of this study are as following:

1. Most common injuries during DMB are upper extremity injuries.
2. Fractures during DMB-injuries are more often than ligament or joint injuries.
3. Small soft tissue injuries occur during upper and lower extremity injuries.
4. Severe injuries are injuries of the a commotio or contusion of the head, as well as thorax injuries.

### Demographics: sex and age

DMB has shown significant growing popularity in the past several years. There is only a small amount of literature showing the profile of downhill mountain bike injuries in recreational athletes^5,8^. Most of the studies focused on injuries during competitive off-road events or in professional athletes^5,6,9,10^. There are some questionnaire-based studies reporting injuries in 50–90% of the participants^1,11^. Besides questionnaires, there are some published case series or case reports, as well as some reviews about the epidemiology of injuries^12–14^. The first prospective study about competitive off-road mountain bikers was published in 1996 by Kronisch et al.^6^, which focused on injury rates, types, and the mechanisms of injuries. As far as we know, we are the first researchers to analyze the incidences and types of injuries in non-professional mountain bike athletes.

Jeys et al.^15^ described an increasing risk of severe injuries in off-road mountain biking due to its increasing popularity across different levels of bikers. A recently published retrospective study involving questionnaires showed a significantly higher incidence of injuries among beginners compared to advanced riders^16^. However, the injuries among advanced riders are more severe, suggesting they take greater risks during riding or even pay less attention to safety equipment^16^. Other studies did not find any differences in the severity of injuries between recreational athletes and elite riders but showed a significantly higher exposure time per year among professional athletes, which leads to higher risk for injury^17^. In 2013, Becker et al.^18^ reported that a significantly higher rate of injuries is reported during competitive downhill mountain biking than during practice, suggesting the higher level of risk taking among athletes during competitions.

Jeys et al.^15^ examined sex differences in off-road mountain bike racing and concluded that there is a higher risk of injuries among women than in men. In our results most of the described patients are male (89.9%). While we do not have data about the absolute rides of men and women, we cannot conclude that women are at higher risk for injuries than men. Recently published studies reported that in injuries among professional athletes, there is a predominance of males in cycling^2,5,19,20^. Kronisch et al.^20^ showed that, overall, even with the higher prevalence of men in off-road cycling competitions, the injury rate is higher for women: 0.77% of women suffered an injury, while only 0.4% of the men did (*p* = 0.01). Aitken et al.^8^ also described an overrepresentation of men between 20 and 29 years of age. In our results, we showed a mean age of 28 overall, with men at the median age of 27.6 years and women at 30.5 years. The possible reasons for more men being injured at the median age of 28 could be their more aggressive riding and willingness to take more risks during downhill riding^8^.

### Mechanisms of injuries

The most common mechanism of injury is described as falling forward (64.9%), and 85.6% of those occurred while riding downhill^5^. Chow et al. described more severe injuries following forward falling, leading to a higher Injury Severity Score (ISS). The mechanisms that particularly lead to severe injuries during mountain biking should be investigated in more detail in future studies in order to better understand the injuries and prevent them.

### Soft-tissue injuries

Overall, in our patient collective, soft-tissue injuries and fractures constituted the most common injuries at 35.4 and 35.6%. In more professional athletes, fractures, head injuries, and joint dislocations constitute the highest group of injuries at 37%^6^. The different characteristics can be explained by the different levels of the participants. Recreational athletes show a different injury characteristic than professional athletes, as professional athletes ride with more speed and take more risks^6^.

A comparative study by Kotlyar et al.^21^ described soft-tissue injuries as the most common injuries. They performed a retrospective analysis including injured cyclists treated in a mountain resort-based medical center during a 3-year study period. They reported lacerations and abrasions of the skin as being the most common injury at 64%, followed by upper extremity fractures at 26%. Our findings show comparable results with small lacerations and abrasions of the skin as the most common soft-tissue injuries. In total, there were 196 small lacerations on the upper extremity and 88 on the lower extremity. Bigger soft-tissue injuries are rare in our cohort. However, soft-tissue injuries often occur in combination with other injuries such as fractures and head injuries^3,5,20^. Since we do not have information about the combination of injuries, we are not able to conclude this from our results.

### Head injuries and thoracic trauma

Head injuries and thoracic trauma were quite rare at 9 and 6%, respectively^21^. Another study published in 2016 from Beck et al.^22^, reporting the injury characteristics of off-road mountain bike riders in Australia, reported spine injuries as the most common and severe injury. Here, 36% of the injuries are described as spine injuries, although a high number of patients also suffered an upper extremity or lower extremity fracture (21%)^22^. Additionally, a retrospective study by Roberts et al.^23^ showed a comparatively high number of head injuries in Canada (56%). They performed a retrospective analysis on severe injuries following mountain biking over a 14-year period. Showing similar results to the Australian study, Roberts et al.^23^ described spinal injuries as the most common severe injury. The findings of these studies differ to our results. Our results show a 7% incidence of head injuries, but only 3% of spine injuries. In our case, spine injuries are rare and are not among the most common injuries. This result could be caused by the trauma center itself, or the downhill conditions being different in the US, Australia, and Canada. Additionally, our study includes a demographic analysis over a period of two seasons. An extended follow-up is necessary with a greater number of patients.

### Fractures and joint injuries

Our patient cohort showed upper extremity fractures as being the most common injury at 59.3%. The second most common injury was the lower extremity fractures at 17.1%. Most studies confirm these findings, with fractures being the most common type of injury that occurs in off-road mountain bikers^2,4,6,20,24,25^. With results similar to ours, previous studies have confirmed that fractures occur most often in the upper extremities, with the clavicle being the most commonly fractured bone^5,15^. In our study, 106 patients in total showed a clavicle fracture, which is almost half of all fractures of the upper extremities (n = 254). Other studies confirm our findings that the radial head (n = 9) and distal radius fractures (n = 52) are the second most common fractures of the upper extremities^15,20,26^.

For joint injuries, AC separation and CC separation are the most commonly reported injuries for off-road mountain bikers^15^. Our findings show the same trend among our patients, with 65 injuries of the AC and CC joint overall. Compared to joint injuries of the upper extremities (n = 116), the lower extremities only show 20 injuries in total, with most of them occurring in the knee (n = 7) or ankle (n = 7). Previous studies show that knee dislocations, leading to injuries of the anterior or posterior cruciate ligament or injuries of the meniscus, often occur among competitive off-road mountain bikers, and shoulder dislocations are also relatively common^1,3,6^. In our cohort, shoulder dislocations were the third most common injury, with 19 shoulder dislocations in total. More studies report the common injury of joint dislocations during off-road mountain biking, but do not specify the joint itself^2,24,27^.

### Severe injuries

There are some studies that report severe injuries following off-road mountain biking that led to death^24,28^. These injuries are described as case reports, where injuries are the result of a missing helmet, or following a collision with the handlebar, causing a rupture of the diaphragm. Most severe injuries in our patient cohort are commotions (n = 35) and contusions (n = 30), accounting for 7% of all injuries in total, followed by one person with intracerebral bleeding and one person with a rupture of the trachea. Such injuries are quite rare and could lead to long-term consequences for the patients. In total, most of the injuries in our patient cohort show a low severity index (NACA-Score), as addressed in Table 2.

### Predictive factors

Some of the predictive factors listed for off-road mountain bike injuries are trail conditions, poor weather, or riding in the dark^29^. Moreover, riders’ characteristics can increase the risk of injuries. Downhill riding at high speed and riders taking more risks lead to a higher risk of injuries overall^3,6^. Recreational riders often ride faster than usual or use unfamiliar bikes, which can increase their risk of injury^30^.

### Limitations

There are some limitations to our retrospective study. First, the data reported are only taken from one mountain bike resort in Austria. The injuries occurred might differ depending on the downhill sections. It may be the case that other mountain bike trails show different injury rates or types depending on the steepness or difficulty of the trail. In addition, the data were taken from the 2020 and 2021 seasons. Due to the COVID-19 pandemic, the number of guests and injuries may have been lower than usual. Moreover, we did not perform any correlations between patients and their experience and fitness level and did not report on their equipment. Especially information about protective gear and a helmet wearing rate, would be of great interest in further investigations on the topic. This information would add to a more precise description of the mechanisms of injuries during DMB.

## Conclusions

DMB is a high-risk sports activity that often leads to injuries of the upper extremities. The most common types of injuries are upper extremity injuries, especially soft-tissue injuries, often in combination with fractures. Severe injuries, such as head injuries or thoraces trauma are rare. In future investigations, it would be of great interest to analyze the data from subsequent years to obtain a better overview of the number of injuries and to determine the mechanisms of injuries. In addition, correlations could be made with predictive factors that lead to injuries, such as the experience and fitness level of the rider, as well as the equipment, which would be interesting to prevent injuries in the future and to provide suggestions for decreasing the risk. A multicenter analysis could be helpful to understand the characteristics and the conditions leading to more frequent and severe injuries.

### Supplementary Information

Supplementary Tables.

## Data Availability

The data presented in this study are available on request from the corresponding author. The data are not publicly available due to privacy.
