# Supplementary material for: Recreational athletes during downhill-mountain biking (DMB) show high incidence of upper extremity fractures in combination with soft-tissue injuries
Source: Sci Rep. 2024 Feb 20;14:4170. doi: 10.1038/s41598-024-54774-7 (PMC10879515; doi:10.1038/s41598-024-54774-7)
Supplement: Supplementary file 1 — Supplementary Tables. [file 41598_2024_54774_MOESM1_ESM.docx]

**Table I Sex Differences of Injury Regions**

|  | | | | | | |
| --- | --- | --- | --- | --- | --- | --- |
|  | | | **Region** | | | Overall |
|  |  |  | Head | Lower extremity | Upper extremity |  |
| **Sex** | Male | Amount | 52 | 97 | 375 | 524 |
|  |  | *% of region* | *85,2%* | *89,8%* | *90,6%* | *89,9%* |
|  |  | *% of total amount* | *8,9%* | *16,6%* | *64,3%* | *89,9%* |
|  | Female | Amount | 9 | 11 | 39 | 59 |
|  |  | *% of region* | *14,8%* | *10,2%* | *9,4%* | *10,1%* |
|  |  | *% of total amount* | *1,5%* | *1,9%* | *6,7%* | *10,1%* |
| Overall | | **Amount** | **61** | **108** | **414** | **583** |
|  |  | *% der Gesamtzahl* | *10,5%* | *18,5%* | *71,0%* | *100,0%* |

Pearson Chi Square=1.664; p=0.435, df=2

**Table II Age Differences of Injury Regions**

|  | | | | | | |
| --- | --- | --- | --- | --- | --- | --- |
|  | | | **Region** | | | Overall |
|  |  |  | Head | Lower extremity | Upper extremity |  |
| Age groups | **<=25 years** | Amount | 33 | 53 | 196 | 282 |
|  |  | *% of region* | *54,1%* | *49,1%* | *47,3%* | *48,4%* |
|  |  | *% of total amount* | *5,7%* | *9,1%* | *33,6%* | *48,4%* |
|  | **26-40 years** | Amount | 19 | 38 | 153 | 210 |
|  |  | *% of region* | *31,1%* | *35,2%* | *37,0%* | *36,0%* |
|  |  | *% of total amount* | *3,3%* | *6,5%* | *26,2%* | *36,0%* |
|  | **>40 years** | Amount | 9 | 17 | 65 | 91 |
|  |  | *% of region* | *14,8%* | *15,7%* | *15,7%* | *15,6%* |
|  |  | *% of total amount* | *1,5%* | *2,9%* | *11,1%* | *15,6%* |
| Overall | | **Amount** | **61** | **108** | **414** | **583** |
|  |  | *% of total amount* | *10,5%* | *18,5%* | *71,0%* | *100,0%* |

Pearson Chi Square=1.071; p=0.899, df=4

[**Table I Sex Differences of Injury Regions**](#_Toc158234381)

[**Table II Age Differences of Injury Regions**](#_Toc158234382)
